# Poly(ethylene oxide)/Ag ions and nanoparticles/1-hexyl-3-methylimidazolium tetrafluoroborate composite membranes with long-term stability for olefin/paraffin separation

**DOI:** 10.1039/c8ra09274e

**Published:** 2019-02-06

**Authors:** Hyunsik Jeon, Sang Wook Kang

**Affiliations:** Department of Chemistry, Sangmyung University Seoul 03016 Republic of Korea swkang@smu.ac.kr +82 2 2287 5362 +82 2 2287 5362; Department of Chemistry and Energy Engineering, Sangmyung University Seoul 03016 Republic of Korea

## Abstract

A poly(ethylene oxide)(PEO)/AgBF_4_/1-hexyl-3-methylimidazolium tetrafluoroborate (HMIM^+^BF_4_^−^) composite membrane that exhibits long-term stability was prepared for olefin/paraffin separation. The membrane was prepared by simply adding AgBF_4_ and HMIM^+^BF_4_^−^ to a solution of PEO. Long-term stability testing showed that the separation performance of the membrane is maintained for ≈100 h owing to the Ag NPs formed in the membrane, which are olefin carriers, being stabilized by HMIM^+^BF_4_^−^. In terms of separation performance, the PEO/AgBF_4_/HMIM^+^BF_4_^−^ composite membrane exhibited a propylene/propane selectivity of 11.8 and a mixed-gas permeance of 11.3 GPU. We also investigated the factors that determine separation performance by comparison with a PEO/AgBF_4_/1-butyl-3-methylimidazolium tetrafluoroborate (BMIM^+^BF_4_^−^) composite membrane. The PEO/AgBF_4_/HMIM^+^BF_4_^−^ composite membrane was characterized by scanning electron microscopy, FT-IR spectroscopy, ultraviolet-visible spectroscopy, thermogravimetric analysis, and Raman spectroscopy.

## Abstract 2

A poly(ethylene oxide)(PEO)/AgBF_4_/1-hexyl-3-methylimidazolium tetrafluoroborate (HMIM^+^BF_4_^−^) composite membrane with long-term stability was prepared for olefin/paraffin separation.

## Graphical abstract

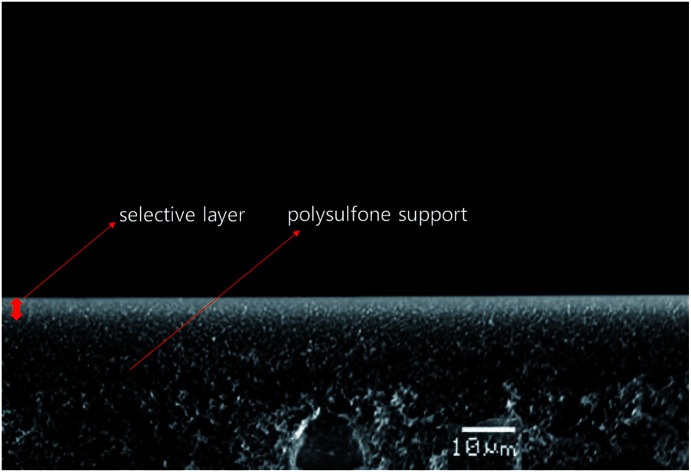

## I. Introduction

Olefins, commonly known as alkenes, are hydrocarbons with carbon–carbon double bonds, while paraffin, known as alkanes, are hydrocarbons having only single bonds.^1^ Light olefins, such as ethylene and propylene, are key monomers in petrochemical and polymer synthesis.^2^ Thus, olefins are used to produce many other organic chemicals. In 2011, 141 million tons of ethylene were produced worldwide.^3,4^ Most olefins are produced by steam cracking of naphtha, propane, and light oil. However, this process produces a mixture of olefins and paraffin.^5^ Therefore, the separation of olefins from olefin/paraffin mixtures is very important in the petrochemical industry.^6–8^ The separation of olefin/paraffin mixtures is most commonly performed by cryogenic distillation because of the similar properties of olefins and paraffin.^9–11^ However, this process suffers from the disadvantages of requiring considerable capital input and being energy intensive.^12^ In order to overcome these problems, membrane separation has emerged as a simple, low-energy manufacturing process.^13,14^ However, while glassy polymer membranes show high selectivity, they typically exhibit low permeability. Conversely, rubbery polymers exhibit high permeability but poor selectivity.^15^ Recently, facilitated transport membranes, which are selectively permeable to only specific substances, have been attracting research attention.^10^ Facilitated transport, which exploits the concentration gradient of solutes generated by the reversible reactions of compounds in membrane with specific solutes, promotes the transport of these solutes.^16^ For example, a dimethylpropylenediamine ethoxyacetate ([DMAPAH][EOAc]) membrane for CO_2_/N_2_ separation using diamine-monocarboxylate protic ionic liquids (PILs) as carriers has been reported to show a selectivity of 151 and a CO_2_ permeability of 3028 barrer.^17^ In addition, an arginine salt-chitosan membrane using the amino group in the arginine salt as a carrier was reported to show a selectivity of 144 and a CO_2_ permeability of 1500 barrer in CO_2_/H_2_ separation.^18^ Recently, it has been reported that PEBAX-1657, which is a permeable polymer that contains Ag ions as olefin carriers, has a propylene/propane selectivity of 8.8 and a permeability of 22.5 GPU.^19^ In general, Ag salts are used as olefin carriers, but Ag ions generated from Ag salts are easily reduced and consequently lose carrier activity.^20–22^ Thus, attempts have been made to partially polarize the surface of Ag NPs so that they react reversibly with olefins.^21^ However, Ag NPs acting as olefin carriers have a drawback in that the permeability of the host membrane is lowered due to the barrier action that occurs when the Ag NPs aggregate to form larger NPs.^22^ In addition, membranes using ZIF and MOF have also been reported.^23–32^ Recently, we fabricated a poly(ethylene oxide)(PEO)/AgBF_4_/1-butyl-3-methylimidazolium tetrafluoroborate (BMIM^+^BF_4_^−^) composite membrane in an attempt to restrict the size of the Ag NPs formed therein. We reasoned that BMIM^+^BF_4_^−^ would stabilize the smaller nascent Ag NPs, inhibiting their aggregation and thus maintaining membrane permeability. The average size of the particles formed in the composite membrane after the long-term test was 4.55 nm. The prepared PEO/AgBF_4_/BMIM^+^BF_4_^−^ composite membrane exhibited a propylene/propane selectivity of ≈15 and a permeability of 12 GPU.^33^ In the current study, we assessed whether stability could be maintained using ionic liquids other than BMIM^+^BF_4_^−^ in order to investigate the factors that contribute to the long-term stability of such membranes. 1-Hexyl-3-methylimidazolium tetrafluoroborate (HMIM^+^BF_4_^−^) was used as the new ionic liquid to investigate the effect of the carbon chain length of ionic liquids, and compared with BMIM^+^BF_4_^−^ in viewpoint of both stabilizing the nanoparticles and separation performance.

## II. Experimental

### 2.1. Materials

Acetonitrile (99.8%) was purchased from Aldrich Chemical Co. and PEO (*M*_w_ 6 × 10^5^ g mol^−1^) was purchased from ACROS Co. Silver tetrafluoroborate (AgBF_4_, 98%) was purchased from Tokyo Chemical Industry Co., and HMIM^+^BF_4_^−^ was purchased from C-TRI. The microporous polysulfone support was provided by Toray Chemical Inc., Korea. All the initial solvents and materials were used without further purification.

### 2.2. Characterization

Scanning electron microscopy (SEM) images were obtained using JEOL JSM-5600LV. The weight loss of the complex was measured using thermogravimetric analysis (TGA, TGA Q50, TA Instruments) under N_2_ flow. The ultraviolet-visible (UV-Vis) absorption spectra were recorded using a Beckman Coulter Life Sciences DU 730 Life Science UV-Vis spectrophotometer with 1 nm resolution. The IR measurements were performed on a VERTEX 70 FT-IR spectrometer; 16–32 scans were signal averaged with a resolution of 8 cm^−1^. Raman spectra of neat HMIM^+^BF_4_^−^ were obtained using a BRUKER RAM II instrument at a resolution of 0.5 cm^−1^.

### 2.3. Membrane preparation

The PEO/AgBF_4_/HMIM^+^BF_4_^−^ complex membrane was prepared by adding AgBF_4_ and HMIM^+^BF_4_^−^ to a PEO polymer solution and vacuum drying. The 5 wt% PEO polymer solution was prepared by dissolving PEO in water/acetonitrile (9 : 10 w/w). AgBF_4_ was added to the prepared PEO polymer solution at 1 : 1 molar ratio, and HMIM^+^BF_4_^−^ was added at different mole ratios. The solution was coated onto polysulfone microporous membrane supports using an RK Control Coater (Model K202, Control Coater RK Print-Coat Instruments Ltd., UK). The long-term stability of the composite membrane prepared at a composition of 1/1/0.052 and vacuum dried for 24 h showed the best performance.

### 2.4. Gas separation performance

The permeation test was performed by permeating the PEO/AgBF_4_/HMIM^+^BF_4_^−^ composite membrane with a propylene/propane mixed gas (1 : 1 v/v). The permeance was measured using a bubble flow meter and the selectivity was assessed by gas chromatography (YoungLin 6500 GC system). The flow rate of the mixed gas was controlled by a mass flow controller (MFC). The unit of gas permeance is GPU (1 GPU = 1 × 10^−6^ cm^3^ (STP)/(cm^2^ s cmHg)).

## III. Results and discussion

### 3.1. SEM images

**Fig. 1:**
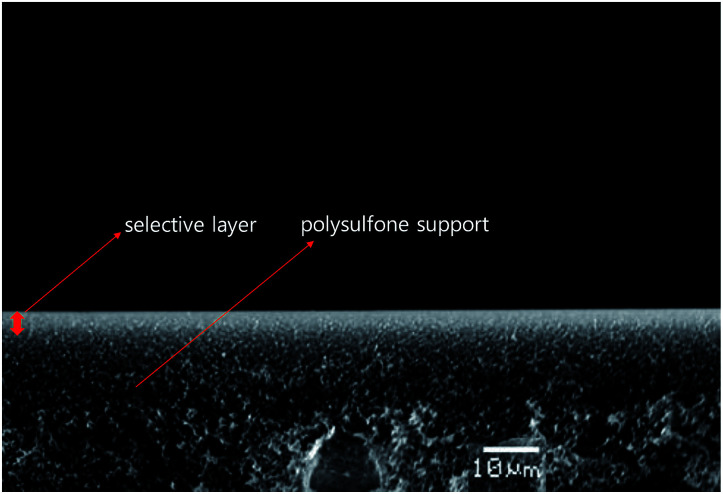
Cross-sectional SEM image of PEO/AgBF_4_/HMIM^+^BF_4_^−^ composite membrane.

Fig. 1 shows a cross-section of the PEO/AgBF_4_/HMIM^+^BF_4_^−^ composite membrane. The membrane was prepared by coating PEO/AgBF_4_/HMIM^+^BF_4_^−^ solution onto a polysulfone support and vacuum drying for 24 h. The sponge-like polysulfone support was well coated with PEO/AgBF_4_/HMIM^+^BF_4_^−^. The thickness of the resultant layer was ≈4.5 μm.

### 3.2. UV-Vis absorption spectra

**Fig. 2:**
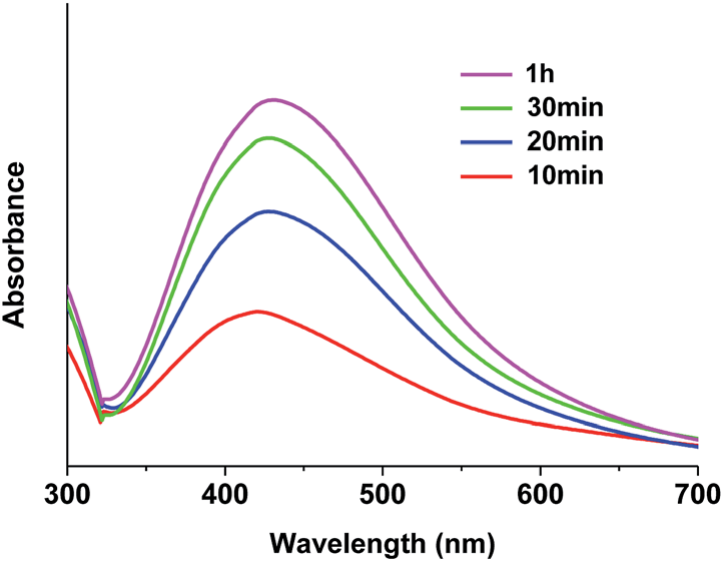
UV-Vis absorption spectra of PEO/AgBF_4_/HMIM^+^BF_4_^−^ solution with heating time.

The stabilizing effect of HMIM^+^BF_4_^−^ on the Ag NPs generated in PEO/AgBF_4_/HMIM^+^BF_4_^−^ solution was investigated by UV-Vis spectroscopy. PEO/AgBF_4_/HMIM^+^BF_4_^−^ solutions were heated at 70 °C for 10, 20, 30, and 60 min. The peak for Ag NPs is typically observed at 420 nm.^21^ Thus, a peak at about 420 nm indicates the formation of Ag NPs, while the intensity of the peak represents the concentration of Ag NPs formed. When the PEO/AgBF_4_/HMIM^+^BF_4_^−^ solution is heated for 10 min, a peak is observed at 403–423 nm. Upon heating for 20 min, a slight peak shift is observed. Upon heating for 30 and 60 min, no further peak shifting is observed, but the peak intensity increases. These results indicate that Ag NPs are stabilized by HMIM^+^BF_4_^−^. This is because the surface of the Ag NPs is partially polarized by HMIM^+^BF_4_^−^, thus inhibiting aggregation and particle growth. As a result, after a heating time of 20 min, only small Ag NPs are formed. Moreover, the spectrum presents a symmetric peak, indicating that monodisperse AgNPs were formed in the PEO/AgBF_4_/HMIM^+^BF_4_^−^ solution. From these results, it was confirmed the effect of stabilizing the NPs by ILs since the relatively narrow peak was observed at UV-Vis spectroscopy (Fig. 2).

### 3.3. FT-IR spectra

**Fig. 3:**
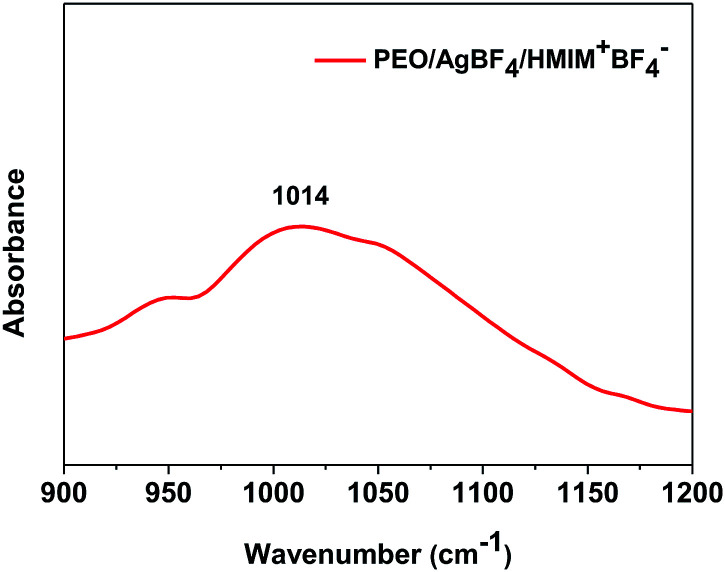
FT-IR spectra of PEO/AgBF_4_/HMIM^+^BF_4_^−^ complex.

FT-IR was measured to confirm the coordination interactions of ether group and Ag ion of PEO. Fig. 3 showed the ether group stretching band peak of PEO/AgBF_4_/HMIM^+^BF_4_^−^ complex. Generally, the ether group stretching band peak of neat PEO was known to be observed at 1082 cm^−1^. When AgBF_4_ was incorporated into PEO, the peak shifted to about 1070 cm^−1^. This was attributable to the interaction between the Ag ion and oxygen in ether group resulting in weaker C–O bonds.^34^ Furthermore, when HMIM^+^BF_4_^−^ was additionally inserted into PEO/AgBF_4_ complex, the peak shifted to 1014 cm^−1^. This could be explained by the interaction of HMIM^+^ with BF_4_^−^ of AgBF_4_, resulting in the weakened bond between Ag ions and BF_4_^−^, and the strong interaction between Ag^+^ and oxygen in ether group. Therefore, the C–O bond became weakened and free BF_4_^−^ of HMIM^+^BF_4_^−^ could stabilize the generated Ag NPs.

### 3.4. Separation performance for propylene/propane mixtures

**Fig. 4:**
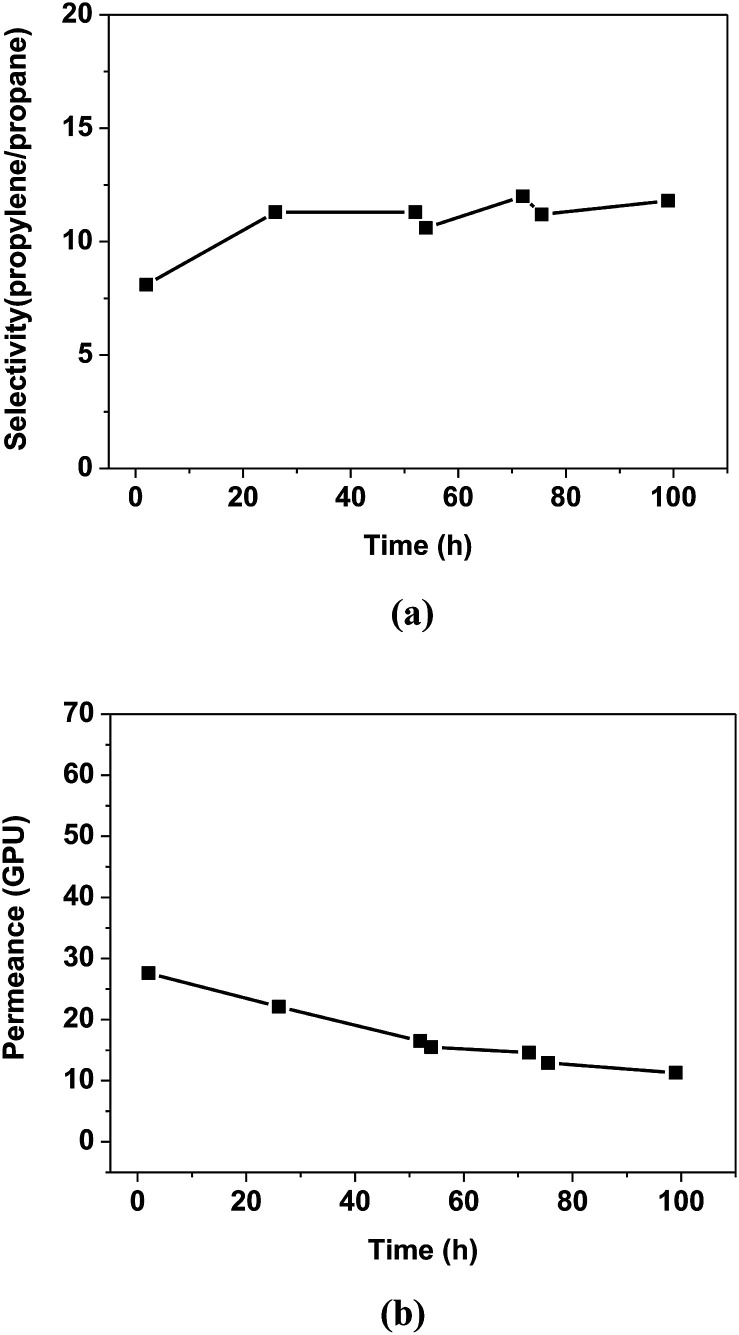
Separation performance of PEO/AgBF_4_/HMIM^+^BF_4_^−^ composite membrane. (a) Selectivity for propylene/propane mixture, and (b) mixed gas permeance.

**Scheme 1:**
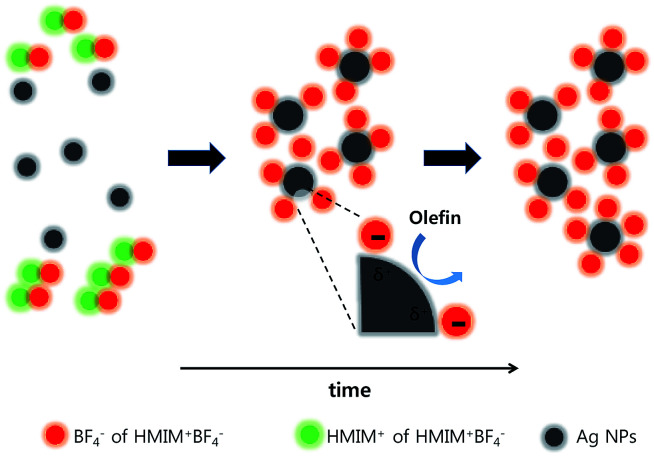
Ag NPs formation in PEO/AgBF_4_/HMIM^+^BF_4_^−^ composite membrane upon heating.

Fig. 4 shows the propylene/propane separation performance of the PEO/AgBF_4_/HMIM^+^BF_4_^−^ composite membrane. The selectivity is initially 8.1 and then increases slightly after 20 h, remaining at ≈11.8 for 100 h. The permeance decreases with the evaporation of the solvent remaining in the membrane, but the rate of decrease slows after 50 h and the permeance remains at 11.3 GPU at 100 h. This separation performance is due to the stabilization of the Ag NPs by HMIM^+^BF_4_^−^. The Ag NPs formed in the PEO/AgBF_4_/HMIM^+^BF_4_^−^ composite membrane are stabilized by HMIM^+^BF_4_^−^, inhibiting their aggregation. Smaller Ag NPs act as more efficient olefin carriers; thus, the propylene/propane separation performance of the PEO/AgBF_4_/HMIM^+^BF_4_^−^ composite membrane is maintained because the NPs do not aggregate as shown in Scheme 1.

### 3.5. Thermogravimetric analysis

**Fig. 5:**
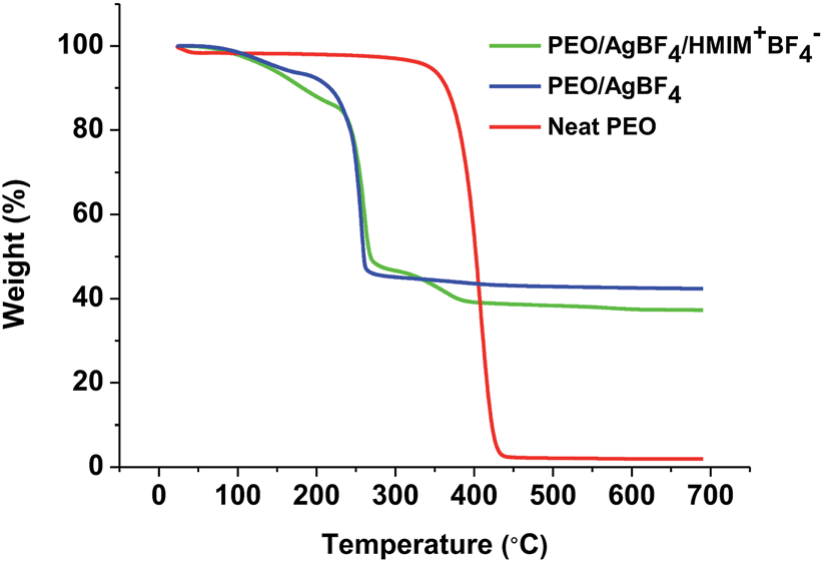
TGA curves for neat PEO (red), PEO/AgBF_4_ (blue), and PEO/AgBF_4_/HMIM^+^BF_4_^−^ (green) complex.

The thermal stabilities of neat PEO, PEO/AgBF_4_, and PEO/AgBF_4_/HMIM^+^BF_4_^−^ membranes were measured by TGA. Fig. 5 shows the weight loss for each membrane at temperatures ranging from room temperature to 700 °C. For the neat PEO membrane, a large weight loss occurs between 380 and 450 °C owing to the degradation of the polymer. The PEO/AgBF_4_ and PEO/AgBF_4_/HMIM^+^BF_4_^−^ membranes exhibit weight losses at lower temperatures owing to the interaction between the added AgBF_4_ and the PEO chains. Furthermore, when HMIM^+^BF_4_^−^ was added to PEO/AgBF_4_, there was significant change in the curve compared to that for PEO/AgBF_4_, indicating that plasticizing effect is produced by the added HMIM^+^BF_4_^−^. This result was same from that for the PEO/AgBF_4_/BMIM^+^BF_4_^−^ composite membrane previously reported. The TGA of PEO/AgBF_4_/BMIM^+^BF_4_^−^ composite membrane was measured in the presence of polysulfone support, but was plasticized like the PEO/AgBF_4_/HMIM^+^BF_4_^−^ membrane.^33^ Due to this plasticizing effect, the permeance does not decrease due to the generation of nanoparticles but the permeance remains constant.

**Fig. 6:**
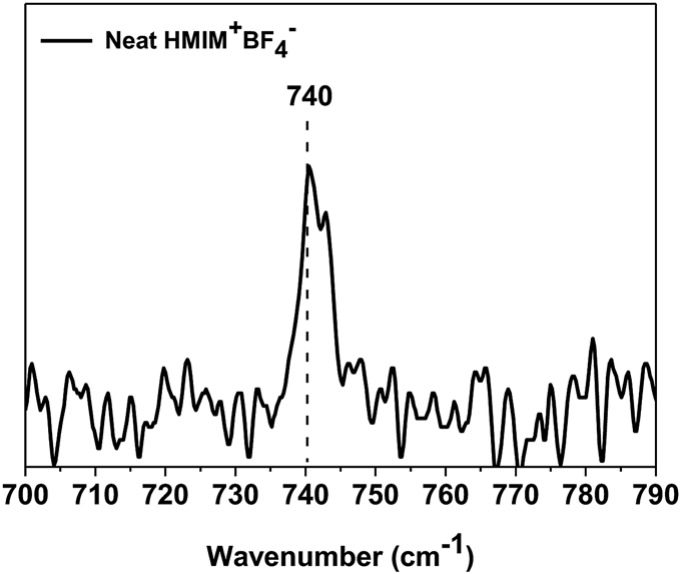
Raman spectrum of neat HMIM^+^BF_4_^−^.

Raman spectra were obtained to investigate the state of BF_4_^−^ in HMIM^+^BF_4_^−^. In general, the free ion, ion pair, and ion aggregate stretching bands of BF_4_^−^ are observed at 765, 770, and 774 cm^−1^, respectively.^35^ In previous studies, the BF_4_^−^ ionic constituents of neat BMIM^+^BF_4_^−^ were found to exist mostly as ion pairs with some ion aggregates.^36^ However, the BF_4_^−^ ionic constituents of neat HMIM^+^BF_4_^−^ are mainly observed to be free ions (740 cm^−1^), as shown in Fig. 6. In general, the more of the ionic liquid that exists as free ions, the better its ability to stabilize Ag NPs. Thus, HMIM^+^BF_4_^−^ stabilizes Ag NPs more than BMIM^+^BF_4_^−^, but HMIM^+^BF_4_^−^ has low molecular mobility because of its large molecular size and thus initially does not stabilize Ag NPs. Therefore, slightly larger Ag NPs are initially formed in HMIM^+^BF_4_^−^. In addition, HMIM^+^BF_4_^−^ molecules do not exhibit good mobility even when they penetrate the polymer, because of their large size. This is consistent with the fact that the plasticizing effect does not occur.

Therefore, the PEO/AgBF_4_/HMIM^+^BF_4_^−^ composite membrane exhibits poorer performance than that of the PEO/AgBF_4_/BMIM^+^BF_4_^−^ composite membrane owing to the increased size of the initially formed Ag NPs and the low mobility of HMIM^+^BF_4_^−^ in the polymer. The separation performances of the PEO/AgBF_4_/HMIM^+^BF_4_^−^ composite membranes were compared in Table 1.

**Table 1:** Propylene/propane selectivity and mixed-gas permeance of PEO/AgBF_4_/HMIM^+^BF_4_^−^ composite membranes after conversion of Ag ions to Ag nanoparticles

	Selectivity (propylene/propane)	Permeance (GPU)
PEO/AgBF_4_/HMIM^+^BF_4_^−^ composite membrane	11.8	11.3

## IV. Conclusions

We succeeded in preparing for the PEO/AgBF_4_/HMIM^+^BF_4_^−^ composite membrane and confirmed the long-term stability of propylene/propane selectivity of 11.8 and permeance of 11.3 GPU for more than 100 h. The long-term stability is owing to the stabilization of the Ag NPs by ionic liquid HMIM^+^BF_4_^−^. HMIM^+^BF_4_^−^ could partially polarize the surfaces of Ag NPs generated under separation process, resulting in the formation of olefin carriers. We also compared the performance of the PEO/AgBF_4_/BMIM^+^BF_4_^−^ composite membrane with that of the current membrane to help elucidate the factors that determine separation performance in such membranes. As a result, the effect of the carbon chain length of ionic liquids was investigated, and both butyl in BMIM^+^BF_4_^−^ and hexyl groups in HMIM^+^BF_4_^−^ were found to be effective in stabilizing the nanoparticles. However, by identifying other ionic liquids with longer carbon chains such as 1-methyl-3-octylimidazolium tetrafluoroborate in the future, we intend to identify main factors in the design of long-term stable facilitated olefin transport membranes.

## Conflicts of interest

There are no conflicts to declare.

## Supplementary Material
